# Comprehensive work up of right ventricular reverse remodelling after successful pulmonary endarterectomy for chronic thrombembolic pulmonary hypertension - a cardiac magnetic resonance study

**DOI:** 10.1186/1532-429X-15-S1-P96

**Published:** 2013-01-30

**Authors:** Andreas Rolf, Stefan Guth, Johannes Boergel, Johannes Rixe, Holger M Nef, Christoph Liebetrau, Sebastian Szardien, Thorsten H Kramm, Grabriele A Krombach, Eckhard Mayer, Christian Hamm

**Affiliations:** 1 Cardiology, Kerckhoff-Heart-And-Thorax-Center, Bad Nauheim, Germany; 2 Thoracic Surgery, Kerckhoff-Heart-And-Thorax-Center, Bad Nauheim, Germany; 3 Radiology, Giessen University, Giessen, Germany

## Background

About 3.8% of patients suffering an acute pulmonary embolism will develop chronic thrombembolic pulmonary hypertension (CTEPH). As a consequence of increasing pulmonary pressure and resistance right ventricular systolic and diastolic function deteriorate while right ventricular volumes increase. Pulmonary thrombendarterectomy (PEA) offers a potential cure with excellent longterm outcome. Cardiac MRI is an ideal tool to monitor remodelling and reverse remodelling before and after PEA, because it allows complete volumetric coverage of the complex right ventricular geometry. Several non invasive parameters which characterize diastolic, systolic and ventriculo-arterial properties can be derived from theses measurements. To quantify the remodelling potential of the right ventricle we performed volumetric measurements before and after PEA

## Methods

Methods: 65 consecutive patients who completed retrospectively ECG gated CINE MRI were included, mean age 41 ± 124 years, 28 female. Volumetric measurements were performed 2 ± 0.5 days pre and 10 ± 2 days post surgery by SSFP Cine sequences (TE: 1.58 ms, TR: 4.8 ms, flip angle: >60°, slice thickness: 8 mm, 2 mm interslicegap) covering the whole right ventricle from apex to base. Ejection Fraction (EF), enddiastolic volume (EDV), endsystolic volume (ESV), stroke volume (SV), diastolic ventricular interaction (left shift of the septum) (DVI) and ventriculo-arterial coupling (Ea-pulm/Es-RV) were derived from volumetric measurements. Right heart catheter measurements in standard procedure were evaluated, if available (retrospective data).

## Results

Results: Pulmonary artery mean pressure and resistance decreased from 46 ± 12 mmHg and 663.2 ± 247.8 dynes * s * cm-5 respectively preoperatively to 31 ± 9 mmHg and 368.1 ± 206.2 postoperatively. EF improved from 26 ± 12% to 44 ± 10% (p = 0.00001), EDV and ESV decreased significantly (EDV 186.3 ± 63.5 to 148 ± 47.2 ml, p = 0.00001; ESV 140.6 ± 61 to 84.8 ± 37 ml, p = 0.00001). DVI with septal left shift was recorded in 80% of patients pre PEA and only 26% of patients post PEA, p=0.00001. Ea-pulm/Es-RV was markedly increased before PEA (3.9 ± 2.8) and significantly decreased to near normal values after surgery (1.4 ± 1.2, p = 0.0001).

## Conclusions

Conclusion: CTEPH before and after PEA is like an on/off phenomenon of pulmonary hypertension and therefore allows to study the reverse remodelling capability of the right ventricle. Cardiac MRI derived parameters reflect changes of function, loading conditions and remodelling. We were able to demonstrate improvement in diastolic properties, ejection fraction, remodelling and loading conditions of the RV in one comprehensive examination. Therefore Cardiac MRI is ideally suited to monitor hemodynamic changes non invasively.

## Funding

No third party funding.

